# Implementing a new model of primary care for stroke survivors living in the community: a mixed-methods process evaluation

**DOI:** 10.1186/s13063-025-08957-w

**Published:** 2025-07-19

**Authors:** Maria Raisa Jessica Aquino, Grace Turner, Elizabeth Kreit, Emily G. Blatchford, Julie Grant, Vicki Johnson, Ricky Mullis, Jonathan Mant

**Affiliations:** 1 Population Health Sciences Institute, Faculty of Medical Sciences, Newcastle University, Newcastle, UK; 2 School of Sport, Exercise and Rehabilitation Sciences, University of Birmingham, Birmingham, UK; 3 National Institute for Health and Care Research BioResource Centre, Cambridge, UK; 4 Primary Care Unit, Department of Public Health and Primary Care, University of Cambridge, Cambridge, UK; 5 University Hospitals Leicester NHS Trust, Leicester Diabetes Centre, Leicester, UK

**Keywords:** Stroke, Primary care, Process evaluation, Intervention fidelity

## Abstract

**Background:**

Survival after stroke is improving, leading to increased demand on primary care and community services to meet the long-term care needs of people living with stroke. Improving Primary Care After Stroke (IPCAS) is a novel model of primary care for stroke survivors living in the community. IPCAS was a complex intervention, with intervention components aimed at stroke survivors and healthcare professionals. This process evaluation aimed to explore how the intervention was delivered in context and how participants engaged with the intervention.

**Methods:**

The process evaluation was underpinned by the National Institute of Health’s Behaviour Change Consortium fidelity framework. A mixed methods design was used to assess fidelity of design, training, delivery and engagement. Methods included questionnaires, video- and audio-recordings, observations, and interviews with staff and participants.

**Results:**

The IPCAS intervention reflected its theoretical underpinnings and was substantially different from usual care. Most components of the intervention were delivered with high or moderate fidelity and training fidelity was high. In particular, the checklist was considered useful. However, other components were less valued, in particular, the direct point of contact service which was hardly used by participants and the local directory of services which had variable take up by healthcare professionals. It was not possible to facilitate communication between primary and secondary care as originally planned. Furthermore, some practices used a GP or healthcare assistant to deliver the reviews, rather than a practice nurse as planned. Several participants did not feel the intervention was relevant for them due to their recovery and the time that had passed since their stroke.

**Conclusions:**

This mixed-methods process evaluation provided novel insights into the design, delivery and engagement with a new model of primary care for stroke survivors. Despite high or moderate fidelity for delivery of most components of the intervention and high training fidelity, some components, such as the direct point of contact, were not valued and several participants did not feel the intervention was relevant for them.

**Trial registration:**

ClinicalTrials.gov NCT03353519. Registered on November 27, 2017.

**Supplementary information:**

The online version contains supplementary material available at 10.1186/s13063-025-08957-w.

## Introduction

Stroke is the second leading cause of death and disability worldwide [1]. From 1990 to 2019, the absolute number of incident and prevalent strokes increased by 70% and 85%, respectively, and are projected to continue to rise [2]. Consequently, there are rising numbers of people with stroke living in the community, and with this comes increasing demand on primary care services to address long-term needs, such as cognitive, psychological and social problems. Primary care services have tended to focus on secondary prevention of stroke and risk factor management rather than addressing the longer-term needs, and no formal primary care-based model of care exists to support community-dwelling stroke survivors [3].

The Improving Primary Care After Stroke (IPCAS) cluster randomised controlled trial (cRCT) aimed to evaluate the clinical and cost effectiveness of a new model of primary care for stroke survivors living in the community compared with standard care [4]. IPCAS was a complex intervention, with intervention components aimed at stroke survivors and healthcare professionals, and was conducted in 23 general practices in the East of England and the East Midlands which delivered this new model of care, and 23 general practices who provided ‘usual care’. Differences between practices may result in varying ways in which the intervention is delivered. Therefore, a multifaceted process evaluation is important to identify successes, failures, unintended consequences and adaptations [5]. Understanding the delivery of complex interventions is key to determining the feasibility of delivering these in other settings and at scale [5]. As such, the present evaluation was developed alongside and conducted in parallel with IPCAS.

The process evaluation aimed to explore (1) how the intervention was delivered; (2) how participants engaged with the intervention; and (3) if/how the context influenced delivery or conduct of the intervention.

## Methods

### Design

This was a mixed-methods process evaluation nested within a cRCT (*n* = 46 general practices; 1040 individual participants). The full protocol for the process evaluation is published elsewhere [6]; methods are summarised below.

The process evaluation was underpinned by the National Institute of Health’s Behaviour Change Consortium fidelity framework [7]. The framework includes five domains: (1) design, (2) training, (3) delivery, (4) receipt and (5) enactment (Table 1).

**Table 1:** Summary of National Institute of Health’s (NIH) Behaviour Change Consortium (BCC) fidelity framework domains, goals, data collection and participants

NIH BCC domain	Goal	Data collection and participants
Design	To determine the extent to which the intervention reflects its theoretical underpinnings and is distinct from ‘usual care’.	Quantitative • Coding intervention components (i.e. IPCAS training manual and MLAS curriculum) to theoretical underpinnings • Comparison of convergence between intervention and control groups (includes recording of participating surgeries’ usual care practices)
Training	To determine the extent to which the training was provided as planned	Quantitative • Training evaluation forms (MLAS) • Video-recorded observations (MLAS) • Audio-recorded observations (IPCAS) • Process variables, such as the number of healthcare professionals and facilitators trained Qualitative • Post-intervention interviews (healthcare professionals, IPCAS)
Delivery	To determine the extent to which stroke survivors understood and applied the skills gained from the intervention	Quantitative • Post-review structured telephone calls to participants (IPCAS) • Self-report questionnaire (MLAS) • Process variables, such as numbers consented, attending reviews and completing MLAS Qualitative • Post-intervention interviews (participants)
Receipt and Enactment	To determine the extent to which stroke survivors understood and applied the skills gained from the intervention	Quantitative• Post-review structured telephone calls to participants (IPCAS)• Self-report questionnaire (MLAS)• Process variables, such as numbers consented, attending reviews and completing MLASQualitative• Post-intervention interviews (participants)

IPCAS, Improving Primary Care After Stroke; MLAS, My Life After Stroke; NIH BCC, National Institute of Health’s Behaviour Change Consortium. 

Favourable ethical opinion was given on 19/12/2017 from Yorkshire & The Humber—Bradford Leeds NHS Research Ethics Committee (17/YH/0441). All participants provided informed consent either in writing or by telephone.

### Intervention and logic model

**Fig. 1:**
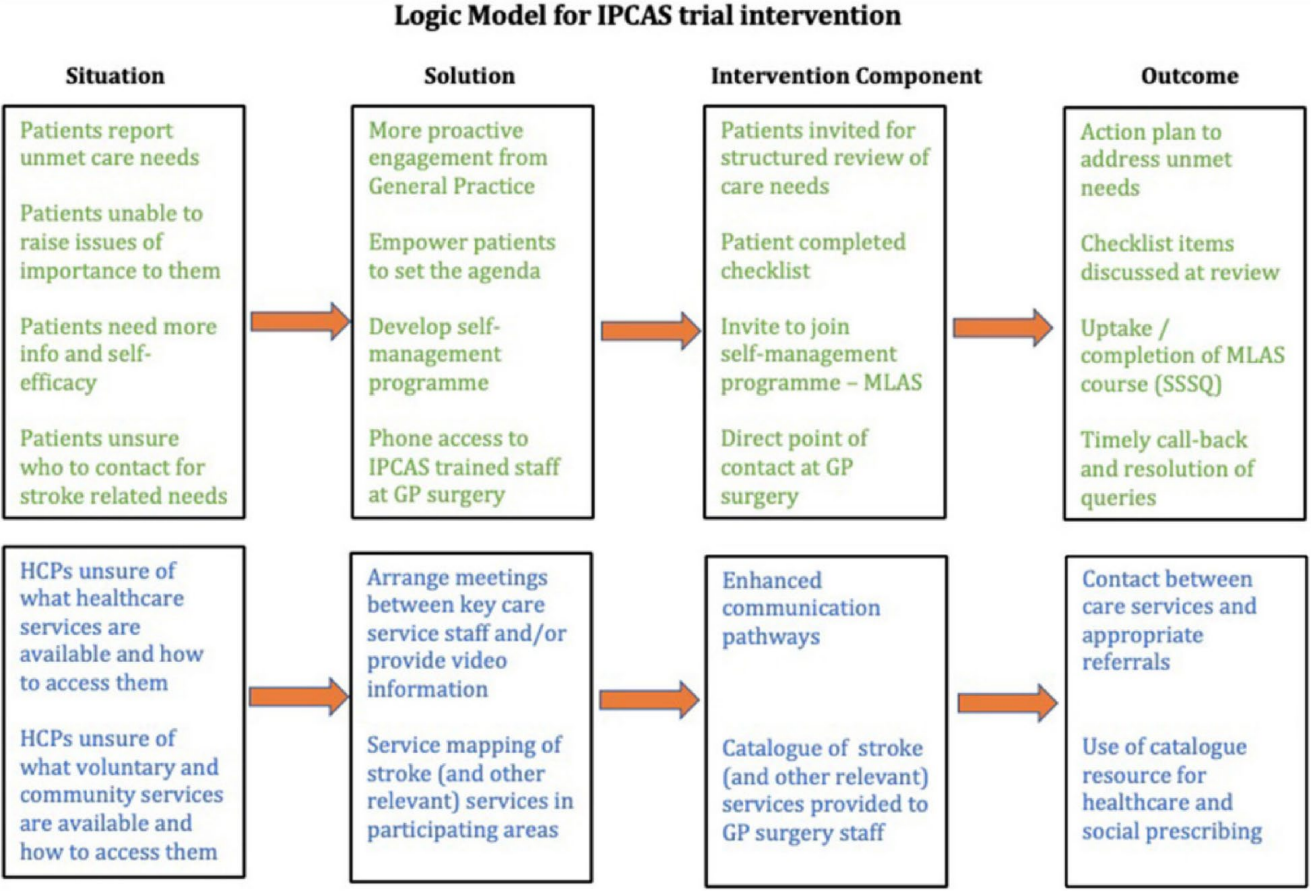
Logic model for the IPCAS trial intervention. GP, general practitioner; HCP, healthcare practitioner; IPCAS, Improving Primary Care After Stroke; SSSQ, Southampton Stroke Self-management Questionnaire

The intervention is summarised below, the full protocol for the IPCAS RCT is published elsewhere [4]. The logic model is depicted in Fig. 1.

The IPCAS model of primary care for stroke survivors living in the community was a multifaceted intervention comprising: a structured review; a direct point of contact; improving interprofessional communication; local service mapping; training of primary care professionals; and self-management programme (‘My Life After Stroke’ [MLAS]) (Table 2). 

**Table 2:** Summary of the planned intervention

Multifaceted intervention comprising: 1. Structured review (20–30 min): a. Performed by practice nurse as part of the regular annual review; b. 15-item needs checklist completed by participants in advance of the review; c. Routine physical check-up (e.g. blood pressure, medication review); d. Agreed action plan (e.g. referral to specialist support) 2. Direct point of contact: provided signposting to services, advice for stroke specific issues, brief support over the phone and arrange follow-up appointments 3. Improving communication between primary and secondary care: meeting arranged for General Practice staff, representatives from hospital stroke services, and the community neuro-rehabilitation teams 4. Directory of local services: mapped stroke and relevant services in the local area 5. Training primary care professionals: overview of potential needs, vignettes based on needs from checklist, familiarised with directory of local services, discussion on how to embed the direct point of contact role 6. Self-management programme: ‘My Life After Stroke’ (MLAS) for stroke survivors and carers. Comprised an individual session, four weekly group-based sessions and a final individual session delivered by 2 trained facilitators	

### Participants and data collection

#### Design

The intervention components (e.g. structured reviews and MLAS) and parameters (e.g. frequency or dose, mode of delivery and duration) were mapped to theoretical underpinnings. Coding was compared by two independent coders, in accordance with established methods [8, 9]. Adaptations to the intervention were recorded and monitored by the research team throughout the intervention period.

To identify if the intervention was distinct from usual care, convergence between intervention and control groups was compared. To capture usual care, participating general practices were asked to provide details of usual care processes for stroke care and long-term follow-up. Intervention components were classified as fully convergent, partially convergent or unique [9].

#### Training

A random sample of the IPCAS training sessions were audio-recorded and two raters used a checklist to record if planned components were present. Interrater agreement was assessed using Cohen’s kappa (95% CI). MLAS training was video-recorded and coded by one independent rater according to a priori criteria to assess whether planned components were present. Trainees (MLAS facilitators) were asked to complete training evaluation forms to assess their comprehension of MLAS and its method of delivery.

Semi-structured qualitative interviews with healthcare providers conducting structured reviews explored experiences of intervention training.

Process variables such as the number of healthcare professionals and facilitators trained, and the duration of training sessions were documented and summarised.

#### Delivery

For the IPCAS intervention, a sample of structured stroke reviews from each general practice were audio-recorded and coded by two independent raters according to a pre-specified checklist. Inter-rater agreement was assessed using Cohen’s kappa (95% CI). In addition, regular structured telephone calls with healthcare professionals were used to monitor intervention delivery, with responses recorded on a pre-specified checklist. Qualitative interviews were conducted with a purposive sample of healthcare providers to explore their experience of delivering the intervention or participation in the study (control).

For the MLAS courses, a sample of sessions were observed. We aimed to observe two of each group session and as many facilitators as possible. Two observers used a bespoke checklist to code the course contents, materials and facilitator behaviours. MLAS facilitator–participant interactions were observed using the Diabetes Education and Self-Management for Ongoing and Newly Diagnosed (DESMOND) observation tool [10].

Process variables were also collected, such as practice list size and role of healthcare professional conducting reviews.

#### Receipt and enactment

As receipt and enactment encompass participant engagement, both were assessed concurrently.

Structured telephone calls were conducted with participants to discuss the structured reviews. Predetermined questions concerning the structured review were recorded on a checklist.

For the MLAS courses, a purposively selected sample of participants were invited to complete a ‘receipt’ questionnaire at the end of each session.

Semi-structured qualitative interviews with participants explored their understanding and experience of the new model of care (intervention arm), and their care experiences since involvement in the trial (control arm). Participants were selected using a random purposive method and most interviews took place during the 6-month follow-up period. Interviewers were experienced qualitative researchers with no prior relationship to, or knowledge of the participants.

Process variables were collected, including the number of patients invited and consented, demographic characteristics, time since stroke, number of patients attending structured reviews, duration of structured reviews, number of patients completing the checklist of needs, number and content of referrals/signposting to other services, number of MLAS courses conducted, number of participants enrolled in MLAS and number of participants completing MLAS.

#### COVID-19

The IPCAS intervention was delivered before the COVID-19 pandemic struck. Five out of 41 participant interviews were conducted remotely during lockdown in the UK (26th March 2020).

### Analysis

#### Quantitative

Descriptive statistics (mean scores, mean percentages and weighted mean percentages across GP practices, where applicable) were calculated for fidelity assessments and process variables using Microsoft Excel. Components were scored as ‘present’ (2/2), ‘attempted/partially present’ (1/2) and ‘absent’ (0/2). Total scores and percentages were averaged for each practice and a weighted mean calculated across practices. Fidelity scores were categorised as high = 80–100%; moderate = 51–79%; and low =  < 50% [11].

#### Qualitative

All audio-recorded qualitative data was transcribed verbatim. NVivo V.12 was used to support qualitative data management and analysis. Interview data was synthesised using thematic analysis by two researchers experienced in qualitative methods [12]. Analysis included iterative comparison of participant and healthcare professional interview data to understand similarities and differences between intervention providers and recipients. Textual data from checklists and questionnaires were analysed using content analysis.

### Patient and public involvement

People with lived experience of stroke and their carers were involved in the initial conception of the research question and study design, prior to the funding submission. During the study, both the Programme Steering Committee and Intervention Development Group included representation from stroke survivors. Four local stroke groups were consulted during the development of the intervention and gave feedback on all the components. Qualitative interview topic guides were piloted with a stroke survivor and refined with their input.

## Results

### Design

Coding of the intervention components and parameters to the theoretical underpinnings demonstrated the intervention reflected its theoretical underpinnings (Table 3 Fidelity of training scores for IPCAS sessionsSession IDTrainerFidelity of training score (%)*A0130 (93.8)B0132 (100.0)C0131 (96.9)D0230 (93.8)*Scoring: 2 = done, 1 = partially done, 0 = not done (maximum score = 32 from 16 items). The two independent raters (RA, JG) achieved 86% Cohen’s kappa agreement (Prevalence and Bias Adjusted Kappa (PABAK) = 0.72)

**Table 3:** Coding of the intervention components to Normalisation Process Theory (NPT; [13])

NPT domain: construct	Present	Evidence
Coherence: differentiation	Yes	Overview of what intervention practices will need to do for the IPCAS trial • Invite participants to an enhanced stroke review (including sending the 15-item checklist) • Perform structured stroke reviews and record outcomes on the trial specific practice template • Distribute information about the ‘My Life After Stroke’ (MLAS) programme • Provide a DPoC telephone service for stroke survivors and their carers • Attend a one-off 2-h meeting with staff from the community and hospital stroke teams Source of evidence from relevant intervention materials: Page 1 (IPCAS Training manual v0.4)
Coherence: communal specification	Yes	Communication with specialists: • Describe who will be attending (GPs, staff conducting reviews, acute, ESD, community) • Describe the structure and purpose of the communication meeting - To build relationships - To discuss how best to contact each service (e.g. named contact or central number?) - To discuss re-referrals to secondary services • Discuss who from the practice will be able to attend (e.g. PI and/or staff conducting reviews) Page 4 (IPCAS Training manual v0.4)
Coherence: individual specification	Yes	Practice staff pair up with a member of the research team (who will act as the patient) and go through the vignette: • Ask the practice staff to vocalise/discuss how they might approach the situation—What they would they want to know?/How would they ask it? Page 6 (IPCAS Training manual v0.4)
Coherence: internalisation	Yes	• Discuss with the staff how they would normally go about reviewing the needs of a patient - Prompts: Is this a new problem?/Has it recently worsened?/Has the patient seen anyone about it before? Action plan (e.g. consultations with GPs/other services/use the service mapping?) Page 5 (IPCAS Training manual v0.4)
Cognitive participation: initiation	Yes	Content Briefly explain the purpose and structure of the training • All staff will be present for the first half of the training (1 h) • All staff will need to sign the trial paperwork at the end of the first half • Sect. 6, structured review core training, will be delivered only to the staff members who will be conducting stroke reviews. This will last roughly another hour Page 1 (IPCAS Training manual v0.4)
Cognitive participation: enrolment	Yes	• Who will attend the communication meeting and what are the best days/times for them Page 3 (IPCAS Training manual v0.4)
Cognitive participation: legitimation	Yes	Direct point of contact: Make sure the practice admin lead (or equivalent) is aware that they will need to disseminate what is discussed to the rest of the practice team (e.g. reception staff) • Our development work highlighted that stroke survivors often do not know who to contact if they have problems relating to their stroke. The DPoC component aims to address this Page 3 (IPCAS Training manual v0.4)
Cognitive participation: activation	Yes	Resources • DPoC guidance document • Screenshots of the DPoC practice template and completion work instructions • Hard copy directory of stroke services and ‘cheat sheet’ for staff conducting reviews • Electronic version of the service mapping Excel file - Research team to file copies of all of these documents in the ISF Page 3 (IPCAS Training manual v0.4)
Collective action: interactional workability	Yes	• How the practice will operationalise the DPoC/who will be acting as the contact? Page 3 (IPCAS Training manual v0.4)
Collective action: relational integration	Yes	• Talk staff through the Excel spreadsheet and how to search - Research team to ask what format would best suit the practice and where the resources will be kept Page 4 (IPACAS Training manual v0.4)
Collective action: skill-set workability	Yes	• How the practice will operationalise the enhanced stroke reviews and when they are planning to complete them Page 3 (IPCAS Training manual v0.4)
Collective action: contextual integration	Yes	• What format for the service mapping resource is best for the practice and where will it be stored • How stroke reviews are usually done in the practice Page 3 (IPCAS Training manual v0.4)
Reflexive monitoring: systematisation and communal appraisal	Yes	The process of assessing *fidelity of delivery* was a formal way of assessing whether providers were delivering the intervention as intended. This was part of the regular communication between the research team and intervention providers as below: Phone calls with the research team: • The research team would like to call the practice (after every 5 reviews) to give discuss any problems and provide feedback - Research team to discuss which days/times would be best for these calls Page 6 (IPCAS Training manual v0.4)
Reflexive monitoring: individual appraisal	Yes	• Outcomes of the review should be recorded on the practice template Page 5 (IPCAS Training manual v0.4)
Reflexive monitoring: reconfiguration	Yes	Stroke review: • Briefly describe the enhanced stroke review - Research team to record how stroke reviews are usually done in the practice - Research team to record who enhanced stroke reviews will be operationalised Page 4 (IPCAS Training manual v0.4)

#### Convergence between intervention and control conditions

All 46 practices reported on their stroke review practices (i.e. ‘usual care’) before intervention rollout. Most practices (*n* = 44) undertook annual reviews with stroke survivors, and of these, 39 practices conducted reviews in person (*n* = 39). Reviews of stroke-related needs were usually done as part of a multimorbidity review (*n* = 32), and were undertaken by various professionals including GPs, nurses and healthcare assistants. The reviews covered Quality and Outcomes Framework (QOF) indicators (i.e. blood pressure reading, cholesterol and blood tests) as well as smoking status, Body Mass Index, and ‘lifestyle’ behaviours (i.e. diet and physical activity).

There were common components between intervention and control conditions, particularly the following: a structured review and measurement of QOF indicators. These overlapping elements reflect partial convergence between the experimental conditions. The remaining intervention components (i.e. directory of stroke services, direct point of contact, enhanced communication, bespoke training of healthcare professionals, MLAS) were all unique to the intervention condition, i.e. absent from the control condition. We found no evidence of contamination between clusters.

#### Adaptations to the intervention

We observed three adaptations to the intervention. To support improved communication between primary and secondary care, it was intended that the research team would set up face-to-face meetings between the specialist stroke team and general practice staff. However, this proved logistically difficult to arrange due to availability of personnel to attend such meetings in both settings. Therefore, a pragmatic approach was adopted whereby the primary care staff were provided videos of the specialist staff explaining their service and how the practice could contact them.

Originally, it was planned that a practice nurse would deliver the reviews; however, some practices used a GP or healthcare assistant or research nurse external to the practice to deliver the reviews.

Finally, it was planned that there would be a group of MLAS facilitators separate to the research team; however, due to facilitator attrition, members of the research team who were trained in MLAS delivered some of the courses.

### Training

#### IPCAS training sessions

Sixty-three HCPs (24 nurses, 18 GPs, 18 practice administrators, 3 healthcare assistants) were trained from 23 general practices between June 2018 and July 2019. Nineteen training sessions were conducted; sessions lasted between 1 and 2 h with an average of 3 trainees per session.

Four training sessions were audio-recorded (21%; 4/19). Training fidelity was high: 96.1% (SD = 6.0; range = 87.5–100.0) of the 16 planned components were delivered (Table 4 and Additional File Table e1).

**Table 4:** Fidelity of training scores for IPCAS sessions

**Session ID**	**Trainer**	Fidelity of training score (%)*
A	01	30 (93.8)
B	01	32 (100.0)
C	01	31 (96.9)
D	02	30 (93.8)

*Scoring: 2 = done, 1 = partially done, 0 = not done (maximum score = 32 from 16 items). The two independent raters (RA, JG) achieved 86% Cohen’s kappa agreement (Prevalence and Bias Adjusted Kappa (PABAK) = 0.72)

In interviews, trainees reported that training met their needs adequately, giving them the confidence to deliver the structured review.

No, once I’d started obviously the first patient was a bit, er, oh my word, am I doing this right or not. But after a couple I found that all the stuff that I had been taught and I've learnt actually came in useful and it all came flooding back after a couple. [Healthcare assistant]

Perceived benefits of training included having a clear understanding of the research methodology and enhanced knowledge of the heterogeneity of stroke survivors. Furthermore, having ongoing support and knowing the research team was always on hand helped build and maintain confidence.

It was quite an eye-opener for me [...] to see how patients’, you know, experiences were very different...Yeah, yeah [...] their journey post stroke had been very different. [Nurse]

#### MLAS training sessions

Two 3-day training sessions were conducted for MLAS in May 2018 and January 2019. The first session had 13 trainees and the second session had 10 new trainees and 10 trainees from the first session (who attended the course as a refresher). Six facilitators dropped out: three before running any MLAS courses and three after delivering one MLAS course.

All MLAS training sessions from the first session were video-recorded. Training fidelity was high: 87.5% of all planned training components were delivered (88.9% of planned materials; 86.9% of planned content) (Table 5).

**Table 5:** Fidelity of training scores from coded video-recordings*^†¥^

Training day	Materials score (%)	Content score (%)	Day total score (%)
Day 1	36 (81.8)	90 (90.0)	126 (87.5)
Day 2	40 (100.0)	95 (89.6)	135 (92.5)
Day 3	36 (85.7)	60 (79.0)	96 (81.4)

*Scoring: 2 = done, 1 = partially done, 0 = not done

^**†**^Number of items scored: day 1–72 (22 materials; 50 content); day 2–73 (20 materials; 53 content); day 3–59 (21 materials; 38 content)

^¥^Note: 16 items (8 planned content; 8 materials—all from day 3) were excluded from the analysis because video-recorded data were not obtained due to the nature of the session

Evaluation forms: 

Feedback from MLAS training evaluation forms for was positive (*n* = 11 for day 1 and *n* = 13 for days 2 and 3; Additional File: Table e2). The majority of respondents either strongly agreed or agreed for most questions about knowledge of the MLAS curriculum and expected facilitator behaviours.

### Delivery

#### IPCAS

Structured reviews were delivered by 24 healthcare professionals (19 nurses, 1 GP, 3 healthcare assistants, 1 research administrator) from 23 general practices. The median practice list size was 11,168.5 (95% CI 8,775, 14,317).

Thirty-four (8%; 34/421) structured reviews across 17 GP practices were audio-recorded and there were 47 structured phone calls to 25 HCPs across 22 GP practices. Audio-recorded observations found reviews were delivered with moderate fidelity: 68.6% (SD = 8.2; range = 13.9–100.0); whereas structured phone calls found high fidelity: 83.1% (SD = 9.1, range = 41.7–100.0) (see Additional File: Table e3 and e4).

Of nine delivery questionnaire items, audio-recorded observations found one was low fidelity (use of the service mapping tool), five were moderate fidelity and three were high fidelity. In contrast, the structured phone calls found that none were low fidelity; four were moderate fidelity and five were high fidelity (Table 6).

**Table 6:** Fidelity of delivery of structured review average scores per item for audio-recordings and structured phone calls to healthcare providers

	**Audio-recordings (*n* = 34)**		**Structured phone calls (*n* = 47)**	
Delivery questionnaire item^†^	Average score (maximum score per item = 2)*	**Average % score**	Average score (maximum score per item = 2)*	**Average % score**
1a—stroke survivor completed checklist	1.7	87.0	1.3	66.0
1b—discussed up to 3 needs	1.8	91.0	1.7	87.0
3a—discussed action plan	1.4	72.0	1.5	76.5
3b—logged/reviewed actions	1.3	63.0	1.4	70.0
4a—provided MLAS information/leaflet	1.7	84.0	1.8	91.5
4b—provided instructions for accessing MLAS	1.4	69.0	1.9	94.5
5a—explained Direct Point of Contact service	1.2	60.5	2.0	99.0
5b—provided instructions for accessing Direct Point of Contact	1.1	56.0	2.0	99.0
6—used service mapping tool	0.7	35.5	1.3	65.0

^†^Note: 2a–2c excluded because they were optional items

*Scoring: 2 = yes, 1 = unsure, 0 = no (maximum score = 18 from 9 items)

Interviews generated three themes relating to HCPs’ experience of delivering IPCAS and factors influencing delivery.

HCPs experience of delivering IPCAS.

The needs checklist was considered useful as it focused conversation, thereby optimising consultation time, and facilitated patient-centred consultation by encouraging stroke survivors to openly discuss needs and enabled them to identify issues important to them.

… with them coming in with that pre- filled in it really did focus one’s mind on areas that were obviously important to the patient. So yeah, it was good. [General practitioner]

However, some HCPs found the checklist too lengthy.

HCPs had mixed views about the usefulness of the service mapping tool. Some found it too lengthy and complex; however, others reported that, after familiarisation, it became easier to use.

Yes, that was as clear as mud! That was a very long list of…that definitely could be improved […] Yeah, and it was very small print, and it was very, kind of, you really were having to, you know, it…yeah […] The print was small, there was too much on one…was it a piece of paper or was it a screen? [Practice nurse]

HCPs reported that they had received clear instructions on how to deliver the direct point of contact; however, all but one participant reported that they had not knowingly received any communications from the stroke survivors using this service. Practice staff perceived that a lot of effort was put into setting up the direct point of contact service.

None of the patients had made any contact with us on the direct point of contact, because we have a special template on for that, and all the staff were educated as to what to do if a patient who is on the IPCAS trial did phone in and needed to speak to either myself or the nurse who was involved, and we’ve not had one phone call about that. So that was interesting. [General practitioner]

HCPs varied in how they introduced MLAS to stroke survivors; some HCPs just handed out the MLAS leaflet, whereas others explained the course in detail.

Action planning, whether it was providing further information/advice or making onward referrals to health and social care services, appeared to make little or no difference to how HCPs referred their patients.

I think I did a normal routine referral for somebody to a physiotherapist, and I think I did one to occupational therapy to check the house, but it didn’t change the way I would normally do it. Certainly didn’t make any difference at all. I would have done it if I’d seen the patient ordinarily, but it just came up at the IPCAS meeting that they needed that so I did the referral. [General practitioner]

Factors influencing delivery.

Mostly, the healthcare staff who delivered the IPCAS intervention were based in the general practice. However, in five practices, nurses external to the practice delivered the intervention. These external nurses experienced challenges relating to not knowing the patient beforehand and lack of knowledge of IT hardware and administrational processes. In contrast, where a practice nurse conducting reviews was an integral member of staff within the surgery, there was often an already-established rapport with the patient which led to a smoother delivery.

I think, because I’ve seen these patients or I had seen them for the last five, six years, a lot of information that we were trying to find out, we had already discussed in previous appointments, not maybe as much in depth … [Practice nurse]

Having the clinical autonomy to make referrals make a difference to how an action plan could be executed, which meant some staff were restricted; for example, being reliant upon GPs to refer patients.

...it has got to be through a GP, because a lot of the hospital-based clinics don’t like nurse referrals, so, you know. [Practice nurse]

HCPs who had previously worked in hospital found this experience helped in delivering the IPCAS review. Experience of technology use influenced delivery; for example, HCPs with limited IT experience struggled with the templates, whereas others found them straightforward and easy to use.

### MLAS

Twenty-two MLAS courses were conducted. Six (27.3%; 6/22) MLAS sessions were observed. MLAS fidelity of delivery was high: 86.4% (SD = 5.4, range = 78.1–92.2, 95% CI = 81.6, 89.2) (Table 7).

**Table 7:** Fidelity of delivery of MLAS (observations)

Course number	Session observed	Raw fidelity of delivery score/maximum score	% score
18	Group session 1	82/96	89.1
24	Group session 1	75/96	78.1
12	Group session 2	93/102	92.2
37	Group session 2	90/102	88.2
18	Group session 3	80/98	81.6
22	Group session 4	91/102	89.2

### Receipt and enactment

#### IPCAS

Of 522 intervention participants, 80.7% (421/522) attended the structured review. Reviews lasted 27.9 min on average (range 16.9–39.4) (see Additional File 1: Table e5).

For the checklist (see Additional File 1, Table e6) and action plan, there were data available for 93.4% (393/421) participants from 22 GP practices.

56.3% (237/421) of participants had at least one action plan recorded. Across 22 practices, there were 431 recorded action plans, split into: 29.5% (127/431) follow-up appointments, 25.3% (109/431) referrals, and 45.2% (195/431) advice (see Additional File 1, Table e7).

67 (15.9%; 67/421) participants from 22 GP practices completed a fidelity of receipt questionnaire by telephone. 63.2% of structured review components were reported to be received by participants, which indicate moderate fidelity (see Additional File 1: Table e8). Of the nine receipt questionnaire items, two were low fidelity; five were moderate fidelity; and two were high fidelity (Table 8).

**Table 8:** Fidelity of receipt of structured review average scores per item (*n* = 67 participants across 22 practices)

Receipt questionnaire item^†^	Average score (maximum score per item = 2)*	Average % score
1—attended structured review	2.0	98.5
2a—completed 15-item checklist	1.7	87.0
2b—discussed up to 3 needs	1.4	69.5
4a—discussed action plan	1.0	51.0
4b—opportunity to review/note agreed action plan	1.0	50.0
5a—received MLAS information/leaflet	1.6	79.0
5b—received instructions for accessing MLAS	1.4	72.0
6a—received information about Direct Point of Contact	0.7	35.5
6b—received instructions on accessing Direct Point of Contact	0.6	31.0

*Scoring: 2 = yes, 1 = unsure, 0 = no

^†^N.B.: Items 3a–c excluded from the analysis because they were optional items

Qualitative interviews were conducted with 19 intervention participants. Four themes were identified: Views and experiences of structured stroke reviews; Perceptions of eligibility for stroke care or support influential to engagement; Engagement with other intervention components, materials and resources; and Benefits gained from participation in IPCAS.

Views and experiences of structured stroke reviews.

Stroke survivors had mixed opinions of the value of structured stroke reviews. Some intervention participants reported feeling cared for and that reviews addressed issues they might not have associated with their stroke (e.g. daily living activities, exercise). In contrast, several stroke survivors could not distinguish the IPCAS structured stroke review from other contacts with the GP practice, owing to high volume of attendance in primary care for other health issues, or impaired memory. Barriers to attendance at the structured stroke review were work commitments and identifying as well-recovered from their stroke.

I was invited to go to the GPs, but the days that they…because they wanted to do an interview there, and I’d had to say, I’m more than willing to do the interview, but I work fulltime, I physically can’t get there to do that at the moment. (Female, 35 years old, 10 years 11 months post-stroke, East Midlands)

Perceptions of eligibility for stroke care or support influential to engagement.

Intervention participants who were several years post-stroke found the IPCAS model of care less relevant or useful to them; however, they acknowledged that structured stroke reviews could be beneficial at earlier stages post-stroke.

What most people need, I would imagine, is support when they first come out and then to review it as they go along. (Male, 84 years old, 5 years 4 months post-stroke, Norfolk)

A barrier to attendance/participation in IPCAS activities was participants’ views that they were fit and well, and therefore any support or care offered to them should be allocated to those experiencing severe post-stroke impacts.

I didn’t think I was serious enough. I thought I was so fit I thought I’d be taking up space for somebody else. (Male, 77 years old, 1 year 6 months post-stroke)

Participants emphasised the importance of providing individualised or tailored support. Some intervention participants reported the 15-item checklist questions did not match their age or experience of stroke (i.e. not severely affected/recovered fully).

Engagement with other intervention components, materials and resources.

Many participants did not recall receiving information about the direct point of contact and, of those that did have an awareness, none accessed it as they felt it unnecessary. Many reported the direct point of contact was a good idea in principle; however, others thought it was superfluous given the availability of other services such as 111 or participants having an established relationship with their GP surgery.

Participants who completed the checklist found it useful for identifying needs and an opportunity to share their experience or symptoms with a healthcare professional, which they had not had opportunity to do so in the past. Participants reported that the checklist enhanced their understanding of their experiences post stroke and address concerns where needed.

Yes, it [15-item checklist] was useful, because it focused me on what has happened to me since or what I’ve found problems, and I think exercise was one of the main things. (Female, 74 years old, 4 years 6 months post-stroke, Ipswich)

Some participants found the tailored service directory (‘service mapping tool’) provided during MLAS useful, specifically information on services that were local to them that they wouldn’t have heard about otherwise.

It [service mapping tool] was very helpful, especially when they gave […] us a sheet of different organisations to try and help. One was to do with transport. (Male, 61 years old, 6 years 4 months post-stroke, Norfolk)

Benefits gained from participation in IPCAS.

Participants found that through engaging with intervention components such as the 15-item checklist of needs and MLAS, they deepened their knowledge and understanding of stroke impacts, increasing their confidence to seek support where needed.

I feel that now, albeit it is a while afterwards, if there was a problem and I thought it was connected, I feel a lot more confident in ringing the doctors and not having to explain myself. (Female, 65 years old, 4 years 2 months post-stroke, Norfolk)

Concerning action planning, some participants reported being offered and accepting extra support or referrals (e.g. memory assessment, physiotherapy) to services at the stroke review.

One thing…yes, he did suggest [at the stroke review] that I had a memory test, and he wasn’t available to do it, he said I could see one of his colleagues and I did go for that. (Female, 74 years old, 4 years 6 months post-stroke, Ipswich)

### MLAS

420 participants were invited to an MLAS course, but only 139 participants took part, of whom 102 completed it. However, those that did attend appeared to value it. Participant data on receipt and enactment of the MLAS self-management programme are reported elsewhere [14].

## Discussion

The IPCAS intervention reflected its theoretical underpinnings and was substantially different from usual care as it focused on psycho-social and physical needs identified by patients, rather than just secondary prevention. Overall, most components of the intervention were delivered with high or moderate fidelity and training fidelity was high. In particular, the checklist was considered useful. However, other components were less valued, in particular, the direct point of contact service which was hardly used by participants and the local directory of services which had variable take up by HCPs. It was not possible to support improved communication between primary and secondary care as originally planned. Furthermore, some practices used a GP or healthcare assistant to deliver the reviews, rather than a practice nurse as planned. Several participants did not feel the intervention was relevant for them due to their recovery of the time that had passed since their stroke.

### Implications for clinicians, policymakers and future research

The RCT findings showed no evidence of any effect of the IPCAS intervention or the MLAS component, on any measured aspect of health status of participants [15]. The process evaluation suggests that the intervention was largely implemented as intended; therefore, lack of effect of the intervention is unlikely to be related to overall fidelity. Good intervention fidelity was achieved through the development of a robust training programme, e.g. provision of curriculum, booster training and mentorship, which minimises the risk of slippage from the intervention being delivered as intended.

This process evaluation taken together with the results of the trial strongly suggests that a ‘blanket approach’ primary care model targeted at all people on stroke registers may not be appropriate. These need to be considered in future intervention designs and effectiveness testing, such that those that have shown promise are replicated, and those less so, adapted or discarded as is appropriate to the relevant setting or context. These are described in detail in the following sections.

Our qualitative data found that the intervention was perceived as less relevant or useful to people who were several years post-stroke and those with good recovery. Similarly, a process evaluation of a long-term care model for stroke patients in the Netherlands found that stroke care co-ordinators considered that some patients had no further need for follow-up care and that they ended the care before their intended 18-month follow-up period [16]. Therefore, our findings suggest that stroke reviews should be time-limited and only continue in selected patients. This is in contrast to guideline recommendations which advocate that regular reviews of stroke survivors are offered in the long term [17]. Audits of guideline implementation suggest that uptake of this recommendation is poor, and this has been interpreted as inadequate service provision [18]. An alternative perspective is that there is a lack of need/demand. Research is required to inform which patients should be offered post-stroke assessment of need in the community in the longer term, i.e. after 6 months.

The intervention was developed with significant input from stroke survivors, yet in practice, it was found not to be relevant to all participants. It is important that patient and public involvement in intervention development reflects the target population (in this case, people with a history of stroke on general practice stroke registers) and considers diverse symptom severity.

Improving communication between primary and secondary care has been identified as a key priority [3]. In developing the IPCAS primary care model, we carried out focus groups with staff involved in care delivery for stroke from both generalist (primary care) and specialist (acute and community) backgrounds. Participants reported silo-based working and strongly endorsed the need for better communication between primary and secondary care [19]. Dissatisfaction about accessibility of and collaboration with GPs was highlighted by a process evaluation of a follow-up care model for stroke patients in the Netherlands [16]. However, we underestimated the difficulties of incorporating improved primary-secondary care communication into our model. Despite being tested in a feasibility study, it was logistically not possible to support improved communication between primary and secondary care through face-to-face meetings between the specialist stroke team and general practice staff at scale. Further research is required to explore alternative solutions for this important issue, such as use of the implementation of interoperable electronic systems to support information sharing and increasing capacity (time and resources) to communicate [20].

We demonstrated the 15-item checklist is practicable to be used in primary care and was valued by HCPs, though some patients raised issues about relevance. Consequently, we recommend use of the checklist to facilitate identification and communication of need when appropriate. The 15-item checklist was developed with stroke survivors/carers and HCPs [21] and provides a practical alternative to other stroke needs checklists which are too lengthy for primary care, such as the 35-item Greater Manchester Stroke Assessment Tool (GM-SAT) (mean appointment length 74 min) [22].

The intended aim of the direct point of contact was to provide signposting to further specialist or community services, offer advice for stroke specific issues, give brief support over the phone, and arrange follow-up appointments and, if appropriate, case management. This was considered useful in principle by participants, but hardly used. Since IPCAS was developed, the social prescriber model has been introduced to primary in the UK [23]. Use of a social prescribing link worker would be a feasible way to offer a direct point of contact in the future.

### Strengths and limitations

To our knowledge, this is the first evaluation of an intervention targeted at people with a history of stroke on general practice stroke registers. In contrast, other stroke follow-up interventions are targeted at people for defined intervals following discharge from hospital. A key strength is that this was a multifaceted evaluation of intervention components using mixed-methods. We have demonstrated that it is feasible, and indeed a worthy investment to deliver a process evaluation alongside a sizeable cRCT, underpinned by existing frameworks, which can inform future intervention refinement and scale up [7]. This is of value given that current literature shows ongoing issues with process evaluation particularly those that assess intervention fidelity, and how this is poorly addressed within clinical trials [24]. A limitation is the sample size for each of the components assessed, which was to ensure that burden on patients/HCP was proportionate given the intensity of involvement in intervention. However, this was mitigated by having different data sources (e.g. interview/checklist/questionnaire).

## Conclusion

Conducting a multifaceted process evaluation alongside a large trial was feasible and provided valuable insight into fidelity, implementation and scale up. Most components of the intervention were delivered with high or moderate fidelity and training fidelity was high; however, some components had limited uptake. Several participants did not feel the intervention was relevant for them due to their recovery or the time that had passed since their stroke. Further work is required to explore how to improve communication between primary and secondary care.

## Supplementary information

Below is the link to the electronic supplementary material.

Additional file 1 Table e1: Fidelity of IPCAS training average scores per item (audio-recorded training sessions)
Table e2: Summary of MLAS training evaluation forms
Table e3: Fidelity of delivery structured review overall scores (Coded audio-recordings)
*Scoring: 2= yes, 1= unsure, 0= no (maximum score= 18 from 9 items)
Table e4: Fidelity of delivery of structured review overall scores (Self-report questionnaire)
*Scoring: 2= yes, 1= unsure, 0= no (maximum score= 18 from 9 items)
Table e5: Attendance and duration of structured stroke reviews, by practice
Table e6: Number of participants completing 15-item checklist of needs by practice
Table e7: Action Plans by GP Practice
Table e8: Fidelity of receipt of structured review overall scores (self-reported questionnaire)
*Scoring: 2= yes, 1= unsure, 0= no (maximum score= 18 from 9 items)

## Data Availability

Data are not available on request due to the highly identifiable nature of the dataset.
