# Supplementary material for: Implementing a new model of primary care for stroke survivors living in the community: a mixed-methods process evaluation
Source: Trials. 2025 Jul 19;26:249. doi: 10.1186/s13063-025-08957-w (PMC12276669; doi:10.1186/s13063-025-08957-w)
Supplement: Supplementary file 1 — Additional file 1 Table e1: Fidelity of IPCAS training average scores per item (audio-recorded training sessions) Table e2: Summary of MLAS training evaluation forms Table e3: Fidelity of delivery structured review overall scores (Coded audio-recordings) *Scoring: 2= yes, 1= unsure, 0= no (maximum score= 18 from 9 items) Table e4: Fidelity of delivery of structured review overall scores (Self-report questionnaire) *Scoring: 2= yes, 1= unsure, 0= no (maximum score= 18 from 9 items) Table e5: Attendance and duration of structured stroke reviews, by practice Table e6: Number of participants completing 15-item checklist of needs by practice Table e7: Action Plans by GP Practice Table e8: Fidelity of receipt of structured review overall scores (self-reported questionnaire) *Scoring: 2= yes, 1= unsure, 0= no (maximum score= 18 from 9 items) [file 13063_2025_8957_MOESM1_ESM.docx]

# Additional File 1

## Training

### IPCAS

**Table e1: Fidelity of IPCAS training average scores per item (audio-recorded training sessions)**

| Training questionnaire item | Average score (maximum score per item = 2) | Average % score |
| --- | --- | --- |
| SR 1a – trainers discussed purpose/structure of checklist | 2.0 | 100.0 |
| SR 1b – trainers provided instructions on how to use checklist | 2.0 | 100.0 |
| SR 2a – trainers discussed option of carrying out physical checks in line with QOF | 2.0 | 100.0 |
| SR 3a – trainers discussed action plan | 2.0 | 100.0 |
| SR 3b – trainers provided instructions on how to log/review actions using review template | 2.0 | 100.0 |
| SR 4a – trainers discussed purpose/structure of MLAS | 2.0 | 100.0 |
| SR 4b – trainers provided instructions for how patients can access MLAS | 2.0 | 100.0 |
| SR 5a – trainers provided instructions for offering direct point of contact service | 2.0 | 100.0 |
| SR 5b – trainers discussed how to provide instructions to patients about contacting practice at the end of review | 1.8 | 87.5 |
| DPOC 1a – trainers discussed the service mapping tool | 2.0 | 100.0 |
| DPOC 1b – trainers discussed how to use the service mapping tool | 1.8 | 87.5 |
| DPOC 2a – trainers gave information on structure/scope of DPOC service: giving advice for stroke-specific issues | 2.0 | 100.0 |
| DPOC 2b – trainers gave information on structure/scope of DPOC service: arranging follow-up appointment(s) | 1.8 | 87.5 |
| DPOC 2c – trainers gave information on structure/scope of DPOC service: brief telephone support/case management | 1.8 | 87.5 |
| EC 1a – trainers discussed broad structure of enhanced communication pathways | 2.0 | 100.0 |
| EC 1b – trainers had initial discussion with healthcare professionals about dates for face-to-face meeting | 1.8 | 87.5 |
| DPOC: direct point of contact; EC: enhanced communication; MLAS: My Life After Stroke; QOF: Quality and Outcomes Framework; SR: structured review | | |

### MLAS

**Table e2: Summary of MLAS training evaluation forms**

|  | **Strongly Agree** | **Agree** | **Neither agree nor disagree** | **Disagree** | **Strongly Disagree** |
| --- | --- | --- | --- | --- | --- |
| ***Day 1 (n=11)*** |  |  |  |  |  |
| **Awareness of stroke problems** | 5 (45.5) | 5 (45.5) | 0 (0.0) | 1 (9.1) | 0 (0.0) |
| **Awareness of strategies to manage cognitive problems** | 4 (36.4) | 2 (18.2) | 4 (36.4) | 1 (9.1) | 0 (0.0) |
| **Understand of emotional problems** | 4 (36.4) | 6 (54.5) | 0 (0.0) | 1 (9.1) | 0 (0.0) |
| **Awareness of strategies to support stroke survivors with emotional problems** | 1 (9.1) | 4 (36.4) | 3 (27.3) | 3 (27.3) | 0 (0.0) |
| **Appreciate stroke effects - relationships** | 5 (45.5) | 6 (54.5) | 0 (0.0) | 0 (0.0) | 0 (0.0) |
| **Awareness of strategies that may support stroke survivors manage effects on their relationships** | 2 (18.2) | 4 (36.4) | 4 (36.4) | 1 (9.1) | 0 (0.0) |
| **Awareness communication problems** | 6 (54.5) | 5 (45.5) | 0 (0.0) | 0 (0.0) | 0 (0.0) |
| **Awareness how to communicate with stroke survivors** | 4 (36.4) | 3 (27.3) | 2 (18.2) | 2 (18.2) | 0 (0.0) |
| ***Day 2 (n=13)*** | |  |  |  |  |
| **Aware of MLAS programme aims** | 9 (69.2) | 4 (30.8) | 0 (0.0) | 0 (0.0) | 0 (0.0) |
| **Aware of why MLAS developed** | 9 (69.2) | 4 (30.8) | 0 (0.0) | 0 (0.0) | 0 (0.0) |
| **My role as facilitator: Curious, non-judgemental approach** | 13 (100.0) | 0 (0.0) | 0 (0.0) | 0 (0.0) | 0 (0.0) |
| **My role as facilitator: Support participants explore thoughts/feelings** | 12 (92.3) | 1 (7.7) | 0 (0.0) | 0 (0.0) | 0 (0.0) |
| **My role as facilitator: Avoid giving specific advice** | 13 (100.0) | 0 (0.0) | 0 (0.0) | 0 (0.0) | 0 (0.0) |
| **My role as facilitator: Facilitate group awareness of information/support sources** | 12 (92.3) | 1 (7.7) | 0 (0.0) | 0 (0.0) | 0 (0.0) |
| **My role as facilitator: Promote knowledge sharing between participants** | 13 (100.0) | 0 (0.0) | 0 (0.0) | 0 (0.0) | 0 (0.0) |
| **Understand theories underpinning MLAS** | 7 (53.8) | 6 (46.2) | 0 (0.0) | 0 (0.0) | 0 (0.0) |
| **Understanding of application of theories to MLAS** | 5 (38.5) | 7 (53.8) | 1 (7.7) | 0 (0.0) | 0 (0.0) |
| **Opportunity to ask questions** | 10 (76.9) | 3 (23.1) | 0 (0.0) | 0 (0.0) | 0 (0.0) |
| **Learnt new skills** | 11 (84.6) | 2 (15.4) | 0 (0.0) | 0 (0.0) | 0 (0.0) |
| **Can apply facilitation skills** | 7 (53.8) | 6 (46.2) | 0 (0.0) | 0 (0.0) | 0 (0.0) |
| **Understand importance of quality assurance** | 12 (92.3) | 1 (7.7) | 0 (0.0) | 0 (0.0) | 0 (0.0) |
| ***Day 3 (n=13)*** | |  |  |  |  |
| **Awareness using MLAS curriculum** | 9 (69.2) | 4 (30.8) | 0 (0.0) | 0 (0.0) | 0 (0.0) |
| **Understanding MLAS content** | 9 (69.2) | 4 (30.8) | 0 (0.0) | 0 (0.0) | 0 (0.0) |
| **Understanding MLAS delivery** | 9 (69.2) | 4 (30.8) | 0 (0.0) | 0 (0.0) | 0 (0.0) |
| **Opportunity to see MLAS resources** | 10 (76.9) | 3 (23.1) | 0 (0.0) | 0 (0.0) | 0 (0.0) |
| **Found 'having a go' useful** | 12 (92.3) | 1 (7.7) | 0 (0.0) | 0 (0.0) | 0 (0.0) |
| **Able to plan delivering MLAS** | 8 (61.5) | 5 (38.5) | 0 (0.0) | 0 (0.0) | 0 (0.0) |
| **Confidence in delivering MLAS** | 5 (38.5) | 6 (46.2) | 2 (15.4) | 0 (0.0) | 0 (0.0) |
| **Opportunity to ask questions** | 10 (76.9) | 3 (23.1) | 0 (0.0) | 0 (0.0) | 0 (0.0) |
| **Learnt new skills** | 11 (84.6) | 2 (15.4) | 0 (0.0) | 0 (0.0) | 0 (0.0) |
| **Can apply skills** | 8 (61.5) | 5 (38.5) | 0 (0.0) | 0 (0.0) | 0 (0.0) |

## Delivery

### IPCAS

**Table e3: Fidelity of delivery structured review overall scores (Coded audio-recordings)**

| Practice ID  (n audio-recorded structured reviews) | Average overall score (maximum score = 18)* | Average overall score in % |
| --- | --- | --- |
| 10 (n= 2) | 12.0 | 66.7 |
| 14 (n= 2) | 13.0 | 72.2 |
| 20 (n= 1) | 12.0 | 66.7 |
| 18 (n= 2) | 14.0 | 77.8 |
| 13 (n= 3) | 16.7 | 92.6 |
| 16 (n= 2) | 6.5 | 36.1 |
| 26 (n= 1) | 16.0 | 88.9 |
| 32 (n= 2) | 13.5 | 75.0 |
| 30 (n= 2) | 6.5 | 36.1 |
| 33 (n= 2) | 2.5 | 13.9 |
| 19 (n= 4) | 18.0 | 100.0 |
| 04 (n= 2) | 11.5 | 63.9 |
| 11 (n= 1) | 18.0 | 100.0 |
| 15 (n= 2) | 8.0 | 44.4 |
| 29 (n= 2) | 12.5 | 69.4 |
| 46 (n= 2) | 17.0 | 94.4 |
| 37 (n= 2) | 9.0 | 50.0 |

**Scoring: 2= yes, 1= unsure, 0= no (maximum score= 18 from 9 items)*

**Table e4: Fidelity of delivery of structured review overall scores (Self-report questionnaire)**

| Practice ID  (n times healthcare professional scored) | Average overall score (maximum score = 18)* | Average overall score in % |
| --- | --- | --- |
| 04 (n= 3) | 17.0 | 94.4 |
| 08 (n= 2) | 16.5 | 91.7 |
| 11 (n= 2) | 12.0 | 66.7 |
| 13 (n= 2) | 14.5 | 80.6 |
| 14 (n= 2) | 15.0 | 83.3 |
| 15 (n= 3) | 15.0 | 83.3 |
| 16 (n= 2) | 13.0 | 72.2 |
| 18 (n= 1) | 14.0 | 77.8 |
| 19 (n= 1) | 18.0 | 100.0 |
| 20 (n= 4) | 14.0 | 77.8 |
| 26 (n= 2) | 16.0 | 88.9 |
| 29 (n= 2) | 13.0 | 94.4 |
| 30 (n= 3) | 13.0 | 72.2 |
| 31 (n= 2) | 16.0 | 88.9 |
| 32 (n= 1) | 16.0 | 88.9 |
| 33 (n= 2) | 7.5 | 41.7 |
| 37 (n= 1) | 16.00 | 88.9 |
| 39 (n= 1) | 13.00 | 72.2 |
| 41 (n= 2) | 17.00 | 94.4 |
| 42 (n= 2) | 16.33 | 90.7 |
| 43 (n= 3) | 16.33 | 90.7 |
| 46 (n= 3) | 16.33 | 90.7 |

**Scoring: 2= yes, 1= unsure, 0= no (maximum score= 18 from 9 items)*

## Receipt and enactment

### IPCAS

**Table e5: Attendance and duration of structured stroke reviews, by practice**

| **Site ID** | **Number Consented** | **Number Attended Review** | **%** | **Mean Review Duration (min)** |
| --- | --- | --- | --- | --- |
| 04 | 30 | 21 | 70.0 | 28.4 |
| 08 | 36 | 28 | 77.8 | Not known |
| 10 | 24 | 24 | 100.0 | 21.1 |
| 11 | 21 | 20 | 95.3 | 28.5 |
| 13 | 30 | 25 | 83.3 | 26.6 |
| 14 | 10 | 8 | 80.0 | 39.4 |
| 15 | 28 | 23 | 82.1 | Not known |
| 16 | 15 | 13 | 86.67 | Not known |
| 18 | 28 | 26 | 92.86 | 20.0 |
| 19 | 23 | 21 | 91.30 | 30.2 |
| 20 | 35 | 31 | 88.6 | 17.6 |
| 26 | 22 | 16 | 72.7 | 32.4 |
| 29 | 18 | 17 | 94.4 | 30.3 |
| 30 | 23 | 19 | 82.6 | Not known |
| 31 | 21 | 18 | 85.7 | 29.6 |
| 32 | 8 | 7 | 87.5 | 28.5 |
| 33 | 15 | 12 | 80.0 | 16.9 |
| 37 | 21 | 14 | 66.7 | 30.9 |
| 39 | 22 | 13 | 59.1 | 32.1 |
| 41 | 25 | 18 | 72.0 | 30.6 |
| 42 | 23 | 17 | 73.9 | 27.4 |
| 43 | 21 | 14 | 66.7 | 27.7 |
| 46 | 23 | 16 | 69.6 | 32.1 |

**Table e6: Number of participants completing 15-item checklist of needs by practice**

| Practice ID | n participants attending review (n available data) | Number Completed Checklist (% of available data) |
| --- | --- | --- |
| 04 | 21 (21) | 18 (85.7) |
| 08 | 28 (23) | 7 (30.4) |
| 10 | 24 (24) | 17 (70.8) |
| 11 | 20 (20) | 11 (55.0) |
| 13 | 25 (25) | 17 (68.0) |
| 14 | 8 (8) | 6 (75.0) |
| 15 | 23 (23) | 18 (78.3) |
| 16 | 13 (13) | 12 (92.3) |
| 18 | 26 (26) | 24 (92.3) |
| 19 | 21 (21) | 18 (85.7) |
| 20 | 31 (31) | 31 (100.0) |
| 26 | 16 (15) | 14 (93.3) |
| 29 | 17 (17) | 17 (100.0) |
| 30 | 19 (0) | 0 (0.0) |
| 31 | 18 (18) | 12 (66.7) |
| 32 | 7 (7) | 5 (71.43) |
| 33 | 12 (13) | 5 (38.46) |
| 37 | 14 (12) | 8 (66.67) |
| 39 | 13 (13) | 6 (46.15) |
| 41 | 18 (17) | 14 (82.35) |
| 42 | 17 (17) | 13 (76.47) |
| 43 | 14 (13) | 8 (61.54) |
| 46 | 16 (16) | 10 (62.50) |

**Table e7: Action Plans by GP Practice**

| **Practice ID** | **n participants attending review (n available data)** | **n Participants With no Action Plan (%)** | **n Follow-up Appointments (%)** | **n Referrals (%)** | **n Given Advice (%)** |
| --- | --- | --- | --- | --- | --- |
| 04 | 21 (21) | 12 (57.1) | 2 (1.6) | 4 (3.7) | 4 (2.1) |
| 08 | 28 (23) | 0 (0.0) | 10 (7.9) | 4 (3.7) | 24 (12.3) |
| 10 | 24 (24) | 7 (29.2) | 13 (10.2) | 19 (17.4) | 13 (11.9) |
| 11 | 20 (20) | 8 (40.0) | 8 (6.3) | 5 (4.6) | 9 (4.6) |
| 13 | 25 (25) | 14 (56.0) | 7 (5.5) | 7 (6.4) | 7 (3.6) |
| 14 | 8 (8) | 3 (37.5) | 5 (3.9) | 0 (0.0) | 1 (0.5) |
| 15 | 23 (23) | 13 (56.5) | 9 (7.1) | 0 (0.0) | 6 (3.1) |
| 16 | 13 (13) | 2 (15.4) | 2 (1.6) | 1 (0.9) | 9 (4.6) |
| 18 | 26 (26) | 20 (76.9) | 2 (1.6) | 2 (1.8) | 4 (2.4) |
| 19 | 21 (21) | 4 (19.1) | 5 (3.9) | 14 (12.8) | 24 (12.3) |
| 20 | 31 (31) | 12 (38.7) | 12 (9.5) | 8 (7.3) | 14 (7.2) |
| 26 | 16 (15) | 4 (26.7) | 8 (6.3) | 5 (4.6) | 3 (1.5) |
| 29 | 17 (17) | 10 (58.8) | 2 (1.6) | 2 (1.8) | 5 (2.6) |
| 30 | 19 (0) | 0 (0.0) | 0 (0.0) | 0 (0.0) | 0 (0.0) |
| 31 | 18 (18) | 9 (50.0) | 4 (3.2) | 2 (1.8) | 5 (2.6) |
| 32 | 7 (7) | 2 (28.6) | 3 (2.4) | 3 (2.8) | 2 (1.0) |
| 33 | 12 (13) | 13 (100.0) | 0 (0.0) | 0 (0.0) | 0 (0.0) |
| 37 | 14 (12) | 9 (75.0) | 4 (3.2) | 0 (0.0) | 0 (0.0) |
| 39 | 13 (13) | 10 (76.9) | 3 (2.4) | 0 (0.0) | 0 (0.0) |
| 41 | 18 (17) | 1 (5.9) | 10 (7.9) | 10 (9.2) | 18 (9.2) |
| 42 | 17 (17) | 0 (0.0) | 6 (4.7) | 8 (7.3) | 20 (10.3) |
| 43 | 14 (13) | 0 (0.0) | 2 (1.6) | 7 (6.4) | 15 (7.7) |
| 46 | 16 (16) | 3 (18.8) | 10 (7.9) | 8 (7.3) | 12 (6.2) |

**Table e8: Fidelity of receipt of structured review overall scores (self-reported questionnaire)**

| Practice ID, by randomisation date  (n participants scored) | Average overall score (maximum score = 18)* | Average overall score in % |
| --- | --- | --- |
| 04 (n= 3) | 12.7 | 70.4 |
| 08 (n= 4) | 9.8 | 54.2 |
| 11 (n= 4) | 12.5 | 69.4 |
| 13 (n= 5) | 11.6 | 64.4 |
| 14 (n= 1) | 14.0 | 77.8 |
| 15 (n= 5) | 9.0 | 50.0 |
| 16 (n= 2) | 12.0 | 66.7 |
| 18 (n= 5) | 10.6 | 58.9 |
| 19 (n= 3) | 11.3 | 63.0 |
| 20 (n= 4) | 12.0 | 66.7 |
| 26 (n= 3) | 13.3 | 74.1 |
| 29 (n= 3) | 15.3 | 85.2 |
| 30 (n= 5) | 9.4 | 52.2 |
| 31 (n= 2) | 14.0 | 77.8 |
| 32 (n= 1) | 12.0 | 66.7 |
| 33 (n= 3) | 8.0 | 44.4 |
| 37 (n= 3) | 12.3 | 68.5 |
| 39 (n= 1) | 10.0 | 55.6 |
| 41 (n= 3) | 13.8 | 83.3 |
| 42 (n= 2) | 14.0 | 77.8 |
| 43 (n= 3) | 5.0 | 27.8 |
| 46 (n= 2) | 13.5 | 88.9 |

**Scoring: 2= yes, 1= unsure, 0= no (maximum score= 18 from 9 items)*
